# Help-Seeking to Cope With Experiences of Violence Among Women Living With HIV in Canada

**DOI:** 10.1177/10778012211019047

**Published:** 2021-07-16

**Authors:** Rebecca Gormley, Valerie Nicholson, Rebeccah Parry, Melanie Lee, Kath Webster, Margarite Sanchez, Claudette Cardinal, Jenny Li, Lu Wang, Rosa Balleny, Alexandra de Pokomandy, Mona Loutfy, Angela Kaida

**Affiliations:** 1 Faculty of Health Sciences, Simon Fraser University, Burnaby, British Columbia, Canada; 2 British Columbia Centre for Excellence in HIV/AIDS, Vancouver, British Columbia, Canada; 3 Chronic Viral Illness Service, McGill University Health Centre, Montreal, Quebec, Canada; 4 Women’s College Research Institute, Women’s College Hospital, Toronto, Ontario, Canada; 5 Faculty of Medicine, University of Toronto, Ontario, Canada; 6 Division of AIDS, University of British Columbia, Vancouver, British Columbia, Canada

**Keywords:** violence, help-seeking, women, HIV, community-based research, CHIWOS

## Abstract

Using baseline data from a community-collaborative cohort of women living with HIV in Canada, we assessed the prevalence and correlates of help-seeking among 1,057 women who reported experiencing violence in adulthood (≥16 years). After violence, 447 (42%) sought help, while 610 (58%) did not. Frequently accessed supports included health care providers (*n* = 313, 70%), family/friends (*n* = 244, 55%), and non-HIV community organizations (*n* = 235, 53%). All accessed supports were perceived as helpful. Independent correlates of help-seeking included reporting a previous mental health diagnosis, a history of injection drug use, experiencing childhood violence, and experiencing sexism. We discuss considerations for better supporting women who experience violence.

Ending violence against women is a global public health priority (Garcia-Moreno & Watts, 2011; WHO, 2017). An estimated one in three women worldwide will experience violence in their lifetime (WHO, 2017). In Canada, women are disproportionately represented in police reports reporting experiences of intimate partner violence, sexual assault, and stalking compared to men (Burczycha & Conroy, 2018; Conroy & Cotter, 2017; Perreault, 2015; Sinha, 2013). Acts of violence are not experienced in isolation but are often compounded by multiple forms of oppression driven by social and structural inequities (Russo & Pirlott, 2006). Such inequities include access to stable housing, living in poverty, racism, sexism, engagement in transactional sex (Decker et al., 2016), and colonialism (Barrett & St Pierre, 2011). In Canada, violence against women must be understood within its colonial history. Intergenerational (Bombay et al., 2014) trauma caused by residential schools (Bombay et al., 2014), the Sixties Scoop (Spencer, 2017), and the ongoing systematic attempts to destroy Indigenous identity, language, and culture has created an environment where Indigenous women experience significantly higher rates of intimate partner and domestic violence (Barrett & St Pierre, 2011; Klingspohn, 2018), structural violence (Proulx, 2014), and interpersonal violence (Razack, 2016).

Previous research illustrates a dynamic relationship between HIV and experiences of violence (Li et al., 2014). An estimated 89% of women living with HIV globally report having experienced or feared experiencing violence throughout their lifetime (Orza et al., 2015), with 25% of a Canadian cohort of women living with HIV reporting that they experienced violence in the last 3 months (Logie et al., 2019). HIV-related stigma may increase risk of experiencing violence among women living with HIV, especially when disclosing their HIV status to a partner (Decker et al., 2016; Dhairyawan et al., 2013; Orza et al., 2015; Zunner et al., 2015). At the same time, women who experience intimate partner violence are at higher risk of acquiring HIV (Kouyoumdjian et al., 2013) as perpetrators rarely use condoms in sexual assault (O’Neal et al., 2013), the physical trauma of sexual assault can increase susceptibility to infection (Draughon, 2012), and controlling tactics can limit a woman’s ability to navigate health decisions, such as negotiating condom use (Coker, 2007). In a Canadian cohort of women living with HIV, forced sex was the third most commonly reported mode of HIV acquisition (16.5%), after consensual sex (51.6%), and sharing needles (19.7%) (Logie et al., 2020).

Violence has significant impacts on the lives and health of women (Coker, 2007) including depression (Logie et al., 2019), impeding engagement in sustained HIV care and antiretroviral therapy (ART) adherence (Carter et al., 2017; Hatcher et al., 2015; Schafer et al., 2012; Zunner et al., 2015), deterring active participation in social and health advocacy (Cho et al., 2020; Schafer et al., 2012), and a synergistic relationship between violence, poly-substance use (Carter et al., 2017), and posttraumatic stress disorder (PTSD; Cuca et al., 2019). While this relationship between violence and HIV has been acknowledged and integrated in both community and national action plans (Khan, 2011; Women’s Coalition, 2014), there is a lack of research that investigates how women cope with experiences of violence, including where women seek help, and the perceived helpfulness of such supports.

## Help-Seeking Post Violence

Support for women experiencing violence can be critical in coping with their experiences and mediating potential long-term impacts of experiencing violence (Kaukinen, 2002; Liang et al., 2005; Postmus et al., 2009). However, support is not always available, accessible, or *helpful*. The likelihood of seeking and receiving help is dependent on the perceived need for services (i.e., for an immediate medical concern) and the structural conditions that shape women’s lives and agency (Cho et al., 2020). HIV-related stigma may discourage disclosure to or discussion of violence with health care providers or social groups (Illangasekare et al., 2014). Previous experiences utilizing help—particularly if a support is perceived to be unhelpful or harmful—may deter future help-seeking (Colman et al., 2014). Help-seeking becomes even more complex when we consider the multiple forms of violence that women living with HIV experience throughout their life course (Cho et al., 2020), including violence from health care providers (Green & Platt, 1997; Nyblade et al., 2009) and lateral violence from their wider communities (Gielen et al., 1997).

Therefore, using data from a Canadian community-collaborative cohort study, the purpose of this analysis is to assess the prevalence and correlates of seeking help among women living with HIV who report experiencing violence as an adult, the sources of supports most frequently accessed, and their perceived helpfulness. By understanding the current use and perceived helpfulness of support systems in place for women living with HIV who experience violence, potential policy and programmatic changes to support women can be identified.

## Methods

We used data from the Canadian HIV Women’s Sexual and Reproductive Health Cohort Study (CHIWOS: www.chiwos.ca), Canada’s largest longitudinal, community-based cohort of women living with HIV. Between August 2013–May 2015, CHIWOS enrolled 1,422 self-identified women living with HIV aged 16 years and older (inclusive of cis, trans, and gender nonbinary women) from three Canadian provinces (British Columbia, Ontario, and Quebec) (Loutfy et al., 2016, 2017). CHIWOS is guided by Women’s Social Determinants of Health (Wuest et al., 2002) and critical feminism (Harnois, 2013) frameworks, which highlight the ways in which socially constructed identities intersect with social and structural inequities to impact the way that women navigate their lives, relationships, and overall health, and employs intersectional principles across a woman’s life course (Carter et al., 2015).

CHIWOS operationalizes the *Greater Involvement of People living with HIV* (*GIPA*) (UNAIDS, 1999, 2007) and the *Meaningful Involvement of Women living with HIV* (*MIWA*) (Loutfy et al., 2016) principles by hiring women living with HIV as Peer Research Associates (PRAs), undergoing training in quantitative research methods to collaborate with researchers, clinicians, policymakers, and community partners to guide survey design, recruitment, data collection, analysis, and knowledge translation activities (Kaida et al., 2019). PRAs administered a computer-assisted questionnaire in English or French supported by FluidSurveys^TM^ software. Median study visit completion time was 120 minutes (interquartile range [IQR]: 90–150). Each participant received a $50 honorarium after completing the survey (Loutfy et al., 2017).

### Ethics and Consent

All participants provided written or witnessed verbal informed consent. Ethical approval was granted from all institutional Research Ethics Boards, including Simon Fraser University, University of British Columbia/Providence Health Care, Women’s College Hospital, McGill University Health Center, and participating clinics and AIDS Service Organizations where requested.

### Analytic Sample

Due to the sensitive and potentially emotionally activating nature of survey questions related to violence, participants had the option of completing the violence section with a PRA, skipping the section altogether, or completing it themselves (without the PRA). Our analysis was restricted to participants who completed the violence section and reported experiencing violence as an adult (≥16 years of age).

Violence is defined as experiencing controlling behaviors, physical, sexual, and/or verbal violence (Logie et al., 2019). Experiencing violence was measured as “yes” to any of the following four questions: “As an adult, has someone ever physically hurt you?”; “As an adult, has someone ever insulted, threatened, screamed, or cursed at you?”; “As an adult, has someone ever restricted your actions by controlling where you can go and what you can do?”; and/or “As an adult, has someone ever sexually forced themselves on you, or forced you to have sex?”

### Main Outcome: Help-Seeking to Cope With Experiences of Violence

Help-seeking to cope with experiences of violence was measured by responses to the question: “Did you ever seek help, such as medical treatment, counselling, or social support to cope with the violence?” Response options included all of the time versus some of the time versus none of the time and were subsequently dichotomized into Yes (sought help all/some of the time) or No (none of the time).

### Supports Accessed, Perceived Helpfulness, and Identifying Missed Opportunities

Women were asked about the types of supports they sought after experiencing violence, including: (a) family/friends (including a partner/spouse); (b) peer support (peers/other people living with HIV, peer navigators/peer counselors, peer support groups); (c) support from non-HIV specific community organizations (staff at a women’s center, sexual health center, rape crisis center, community organization, support group for women); (d) religious/spiritual support (traditional healer, Elder, religious counselor); (e) health care providers (doctors, nurses, social workers, mental health counselors); or (f) legal advisors/traditional justice. Women were then asked about the perceived helpfulness of each support accessed by answering the questions, “Of the people and services you consulted, how useful were they in helping you cope with your experience?” Responses were dichotomized as helpful (very helpful or a little bit helpful) or unhelpful (not at all helpful).

Women who did not report seeking help were asked to identify any supports that might have been helpful in coping with their experiences by answering, “Are there supports that you think might have been or might be helpful in coping with your experience?” Responses were grouped as follows: (a) family/friends (including a partner/spouse); (b) peer support (peers/other people living with HIV, peer navigators/peer counselors, peer support groups, peer support group for women); (c) support from non-HIV specific community organizations (staff at a women’s center, sexual health center, rape crisis center, community organization); (d) religious/spiritual support (traditional healer, Elder, religious counselor); (e) health care providers (doctors, nurses, social workers, mental health counselors); or (f) legal advisors/traditional justice.

### Covariates

Covariates of help-seeking to cope with experiences of violence were identified *a priori* based on available literature and informed by the living experiences of women living with HIV advising this study. Covariates included socio-demographic factors, including province of interview (BC vs. ON vs. QC), age, ethnicity (Indigenous vs. African/Caribbean/Black vs. White vs. Mixed Race/Other), legal status in Canada (Canadian citizen vs. landed/permanent resident vs. refugee/other), gender identity (woman vs. Transwoman/Two-Spirited/Queer/Other), sexual orientation (heterosexual vs. lesbian, gay, bisexual, transgender, and queer [LGBTQ]), annual gross household income (<$20,000 CAD vs. ≥$20,000), housing stability (stable vs. unstable), education (lower than high school vs. high school or higher), experiences of violence as a child (No vs. Yes), current sex work (No vs. Yes), ever injection drug use (No vs. Yes), incarceration experiences (Never vs. Ever [but not within the past year] vs. Recent [within the past year]), number of dependents (0 vs. 1 vs. ≥2), and food security (secure vs. insecure). Stable housing was defined as renting or owning an apartment, house, self-contained room in a house or apartment, or living in a group home; unstable housing was defined as a self-contained room with or without amenities, a transition/halfway/safe house, couch surfing, or living outdoors or in a car.

Psychosocial factors included diagnosis of a mental health condition ever (No vs. Yes), resilience [the Resilience Scale RS-10, a 10-item scale scored on a 7-point scale, with higher scores indicating higher resilience (Wagnild & Young, 1993)], experiencing racism [the Everyday Discrimination Scale—racism, with higher scores indicating higher levels of racism (Williams et al., 1997)], experiencing sexism [the Everyday Discrimination Scale—sexism, with higher scores indicating higher levels of sexism (Williams et al., 1997)], and HIV stigma using the 10-item HIV Stigma Scale [ranges: 0–100, with higher scores indicating higher stigma (Wright et al., 2007)]. HIV-related clinical factors included years living with HIV (<6 years vs. 6–14 years vs. >14 years) and current antiretroviral (ARV) use (currently on ARVs vs. not currently on ARVs but previously vs. never on ARVs).

### Statistical Analyses

This analysis used cross-sectional data from the CHIWOS baseline survey. Baseline characteristics of study participants were described for the cohort overall and by province. We used row percentages to report the proportion of help-seeking among women with different identities and experiences seeking help for violence. In bivariable analysis, associations between help-seeking to cope with experiences of violence (yes vs. no) and covariates were assessed using Fisher’s exact test and Pearson’s chi-square test as appropriate for categorical variables or Wilcoxon Rank Sum test for continuous variables. All *p* values are two-sided with a significance level of α < 0.05. Don’t know/prefer not to answer responses were excluded from the *p* value calculations.

Explanatory univariable and multivariable logistic regression (the latter adjusting for factors meeting confounding criteria) were used to investigate factors associated with help-seeking to cope with experiences of violence, using **no** as the reference category. Unadjusted (ORs) and adjusted odds ratios (aORs) with 95% confidence intervals (CIs) are reported. Independent variables in the final model were selected based on Type-III *p* values (based on the Type-III Sum of Squares testing the overall effect of variables) and Akaike Information Criterion (AIC). The variable with the highest Type-III *p* value was dropped at each step of the selection process until the model reached the lowest AIC (lower AIC indicates better model fit). Observations with “don’t know,” “prefer not to answer,” or missing responses were removed. Analyses were conducted using SAS version 9.4 (SAS, North Carolina, United States).

## Results

Of the 1,422 women living with HIV who completed the baseline survey of CHIWOS, women who chose not to complete the violence section (*n* = 105) were excluded. Of the remaining 1,317, 80% (*n* = 1,057) reported experiencing violence as an adult and were included in the analytic sample. A bivariate analysis was performed to assess differences in socio-demographic and psychosocial differences between the women included in the analysis (*n* = 1,057), and the women who were excluded because they did not complete the violence section (*n* = 105). We found a higher proportion of African, Caribbean, or Black women chose not to complete the violence section (*n* = 45, or 14% chose not to complete the violence section) compared to other ethnicities (*p* < .001). Other statistically significant differences were found among women who reported a lower than high school education (*p* = .021), and women with two or more dependents (*p* = .007). The results can be found in Supplementary File 1.

Baseline characteristics of the analytic sample are found in Table 1. The median age was 43 [IQR: 36–50], with 247 (23%) identifying as Indigenous; 273 (26%) as African, Caribbean, or Black; 456 (43%) as white; and 81 (8%) as mixed race or other. The majority identified as cisgender women (*n* = 1,009, 96%), and heterosexual (*n* = 909, 86%). While most women reported living in stable housing (*n* = 943, 89%), over half were food insecure (*n* = 685, 65%) and had an annual income of less than $20,000 CAD (*n* = 681, 64%). Approximately 35% (*n* = 369) had been living with HIV for more than 14 years, and 905 (86%) were on ART at the time of their interview.

**Table 1.** Baseline Characteristics of Women Seeking Help After Experiences of Violence Among Women Living With HIV Enrolled in CHIWOS (*n* = 1,057) (row %).

Variable	Overall (*n* = 1,057) *n* (col %) or median [IQR]	Help-seeking		
		None (*n* = 610) *n* (row %) or median [IQR]	All/some (*n* = 447) *n* (row %) or median [IQR]	*p* value
Province of interview				<.001
British Columbia	316 (29.9)	142 (44.9)	174 (55.1)	
Ontario	472 (44.7)	314 (66.5)	158 (33.5)	
Quebec	269 (25.4)	154 (57.2)	115 (42.8)	
Age	43 [36–50]	43 [36–51]	44 [36–50]	.706
Ethnicity				<.001
Indigenous	247 (23.4)	117 (47.4)	130 (52.6)	
African/Caribbean/Black	273 (25.8)	185 (67.8)	88 (32.2)	
White	456 (43.1)	256 (56.1)	200 (43.9)	
Mixed race/Other	81 (7.7)	52 (64.2)	29 (35.8)	
Legal status in Canada				.003
Canadian citizen	870 (82.3)	482 (55.4)	388 (44.6)	
Landed/permanent resident	111 (1.5)	78 (70.3)	33 (29.7)	
Refugee/other	72 (6.8)	48 (66.7)	24 (33.3)	
DK/PNTA	4 (0.4)	2 (50.0)	2 (50.0)	
Gender identity				.492
Woman	1009 (95.5)	580 (57.5)	429 (42.5)	
Transwoman/two-spirited/queer/Other	48 (4.5)	30 (62.5)	18 (37.5)	
Sexual orientation				.038
Heterosexual	909 (86.0)	535 (58.9)	374 (41.1)	
LGBTQ	143 (13.5)	71 (49.7)	72 (50.3)	
DK/PNTA	5 (0.5)	4 (80.0)	1 (20.0)	
Household yearly income				.038
<$20,000	681 (64.4)	376 (55.2)	305 (44.8)	
≥$20,000	352 (33.3)	218 (61.9)	134 (38.1)	
DK/PNTA	24 (2.3)	16 (66.7)	8 (33.3)	
Housing stability				.966
Stable	943 (89.2)	544 (57.7)	399 (42.3)	
Unstable	114 (1.8)	66 (57.9)	48 (42.1)	
Education				.057
Lower than high school	178 (16.8)	91 (51.1)	87 (48.9)	
High school or higher	875 (82.8)	515 (58.9)	360 (41.1)	
DK/PNTA	4 (0.4)	4 (100.0)		
Experienced violence as a child				<.001
No	212 (2.1)	157 (74.1)	55 (25.9)	
Yes	836 (79.1)	446 (53.3)	390 (46.7)	
DK/PNTA	9 (0.9)	7 (77.8)	2 (22.2)	
Current **s**ex work				.630
No	938 (88.7)	545 (58.1)	393 (41.9)	
Yes	76 (7.2)	42 (55.3)	34 (44.7)	
DK/PNTA	43 (4.1)	23 (53.5)	20 (46.5)	
Injection drug use (ever)				<.001
No	651 (61.6)	423 (65.0)	228 (35.0)	
Yes	390 (36.9)	173 (44.4)	217 (55.6)	
DK/PNTA	16 (1.5)	14 (87.5)	2 (12.5)	
Incarceration				<.001
Never	596 (56.5)	393 (65.9)	203 (34.1)	
Ever (but not last year)	384 (36.4)	174 (45.3)	210 (54.7)	
Recent (within the last year)	74 (7.0)	42 (56.8)	32 (43.2)	
Number of dependents				.408
0	611 (57.8)	354 (57.9)	257 (42.1)	
1	206 (19.5)	111 (53.9)	95 (46.1)	
≥2	238 (22.5)	143 (60.1)	95 (39.9)	
DK/PNTA	2 (0.2)	2 (100.0)		
Food security				.022
Secure	368 (34.8)	230 (62.5)	138 (37.5)	
Insecure	685 (64.8)	378 (55.2)	307 (44.8)	
DK/PNTA	4 (0.4)	2 (50.0)	2 (50.0)	
Psychosocial factors				
Mental health diagnosis				<.001
No	554 (52.4)	384 (69.3)	170 (30.7)	
Yes	493 (46.6)	219 (44.4)	274 (55.6)	
DK/PNTA	10 (0.9)	7 (70.0)	3 (30.0)	
Resilience scale	64 [58–68]	64 [58–69]	63 [57–67]	.025
Everyday racism scale	17 [8–29]	16 [8–27]	19 [8–31]	.025
Everyday sexism scale	19 [11–28]	18 [10–27]	22 [13–29]	<.001
HIV stigma scale	58 [43–70]	58 [43–70]	60 [45–73]	.084
Medical information				
Years living with HIV				.805
Less than 6 years	242 (22.9)	138 (57.0)	104 (43.0)	
6**–**14 years	413 (39.1)	237 (57.4)	176 (42.6)	
More than 14 years	369 (34.9)	219 (59.3)	150 (40.7)	
DK/PNTA	33 (3.1)	16 (48.5)	17 (51.5)	
Current antiretroviral use				.051
Currently on ARVs	905 (85.6)	510 (56.4)	395 (43.6)	
Not currently but previously	53 (5.0)	32 (60.4)	21 (39.6)	
Never on ARVs	97 (9.2)	67 (69.1)	30 (30.9)	
DK/PNTA	2 (0.2)	1 (50.0)	1 (50.0)	

*Note.* IQR = interquartile range; DK / PNTA= don’t know/prefer not to answer; LGBTQ = lesbian, gay, bisexual, transgender, and queer. All percentages were weighted for sample probabilities; therefore, percentages reported in tables represent national estimates.

Overall, 42% (*n* = 447) reported help-seeking to cope with experiences of violence, and 58% (*n* = 610) did not seek help, with a higher proportion of women seeking help in British Columbia (*n* = 174, 55%) compared to Ontario (*n* = 158, 34%) and Quebec (*n* = 115, 43%) (*p* < .001). A higher proportion of women who identified as Indigenous reported help-seeking to cope with experiences of violence (*n* = 130, 53%) compared to African, Caribbean, Black women (*n* = 88, 32%), white women (*n* = 200, 44%), and women who identified as mixed race or other ethnicity (*n* = 29, 36%) (*p* < .001). Similarly, half of the women who identified as LGBTQ sought help (*n* = 72, 50%), compared to 41% of heterosexual women (*n* = 374, 41%, *p* = .038). More women who were food insecure sought help (*n* = 307, 45%) compared to women who were food secure (*n* = 138, 38%, *p* = .022). A lower proportion of women who had never been incarcerated sought help (*n* = 203, 34%) compared to women who had previously (*n* = 210, 55%) and recently (*n* = 32, 43%) been incarcerated (*p* < .001). And women who had ever used injection drugs sought help at a higher frequency (*n* = 217, 56%) than women who had not (*n* = 228, 35%, *p* < .001). A higher proportion of women who had ever been diagnosed with a mental health condition sought help (*n* = 274, 56%) than women who had not (*n* = 170, 31%, *p* < .001). There were no significant differences found between age, gender identity, housing stability, education, engagement in sex work, and engagement in HIV care.

### Explanatory Logistic Regression

Overall, 956 observations were included in the model, after removing observations with “don’t know” or “prefer not to answer,” and any missing observations due to skip patterns in selected variables. Table 2 presents the ORs, aORs, and 95% CIs of help-seeking to cope with experiences of violence by covariates of interest. In univariate analyses, covariates significantly associated with help-seeking to cope with experiences of violence inversely, referent to no, included province of interview; African, Caribbean, Black ethnicity; reporting refugee, or landed/permanent resident status; reporting an annual household income ≥$20,000 CAD; and never having taken ARVS. Accordingly, women who identified as Indigenous, LGBTQ, having previous (but not within the past year) incarceration experience, diagnosis of a mental health condition, history of injection drug use, experienced violence as a child, and experience sexism in their daily lives had higher unadjusted odds of help-seeking to cope with experiences of violence.

**Table 2.** Explanatory Logistic Regression of Factors Associated With Seeking Help After Experiencing Violence as an Adult, in Reference to Never Seeking Help, Among Women Living With HIV in CHIWOS (*n* = 956).

Variable	Seeking help after experiencing violence as an adult	
	OR (95% CI)	aOR (95% CI)
Province of interview		
British Columbia	1.00 (Reference)	1.00 (Reference)
Ontario	0.44 (0.32, 0.59)	0.59 (0.42, 0.82)
Quebec	0.66 (0.47, 0.93)	1.04 (0.71, 1.53)
Age	1.01 (0.99, 1.02)	Not selected
Ethnicity		
White	1.00 (Reference)	
Indigenous	1.39 (1.01, 1.93)	Not selected
African/Caribbean/Black	0.59 (0.42, 0.82)	
Mixed race/other	0.74 (0.44, 1.23)	
Legal status in Canada		
Canadian citizen	1.00 (Reference)	
Landed/permanent resident	0.60 (0.38, 0.93)	Not selected
Refugee/other	0.56 (0.32, 0.98)	
Gender identity		
Woman	1.00 (Reference)	Not selected
Transwoman/two-spirited/queer/other	0.90 (0.48, 1.70)	
Sexual orientation		
Heterosexual	1.00 (Reference)	Not selected
LGBTQ	1.53 (1.05, 2.23)	
Annual household income		
<$20,000	1.00 (Reference)	Not selected
≥$20,000	0.72 (0.55, 0.95)	
Violence as a child		
No	1.00 (Reference)	1.00 (Reference)
Yes	2.39 (1.68, 3.40)	1.81 (1.24, 2.63)
Injection drug use (ever)		
No	1.00 (Reference)	1.00 (Reference)
Yes	2.26 (1.73, 2.95)	1.68 (1.25, 2.28)
Incarceration		
Never	1.00 (Reference)	
Ever (but not last year)	2.13 (1.62, 2.79)	Not selected
Recent (within the last year)	1.52 (0.92, 2.52)	
Mental health condition		
No	1.00 (Reference)	1.00 (Reference)
Yes	2.97 (2.28, 3.87)	2.54 (1.91, 3.39)
Resilience scale	0.99 (0.97, 1.00)	1.01 (1.00, 1.03)
Everyday racism scale	1.01 (1.00, 1.02)	Not selected
Everyday sexism scale	1.02 (1.01, 1.04)	1.02 (1.01, 1.03)
HIV stigma scale	1.01 (1.00, 1.01)	Not selected
Current ARV use		
Currently on ARVs	1.00 (Reference)	
Not currently, previously	0.89 (0.48, 1.63)	Not selected
Never on ARVs	0.62 (0.39, 0.98)	

*Note.* OR = odds ratio; CI = confidence interval; aOR = adjusted odds ratio; LGBTQ = lesbian, gay, bisexual, transgender, and queer. All percentages were weighted for sample probabilities; therefore, percentages reported in tables represent national estimates.

In adjusted analyses, women in Ontario had significantly lower odds of help-seeking (aOR = 0.59 [0.42–0.82]) compared to women in British Columbia. Women who had previously been diagnosed with a mental health condition (aOR = 2.54 [1.91–3.39]), who reported a history of injection drug use (aOR = 1.68 [1.25–2.28]), experiencing violence as a child (aOR = 1.81 [1.24–2.63]), and experiencing sexism in their daily lives (aOR = 1.02 [1.01–1.03]) had higher odds of help-seeking to cope with experiences of violence. Resilience was not significantly associated with help-seeking (aOR = 1.01 [1.00–1.03]).

### Supports Accessed and Perceived Helpfulness in Coping With Experiences of Violence

**Figure 1.**
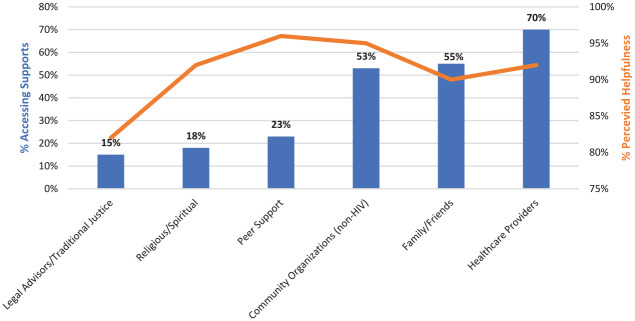
Supports accessed by women living with HIV experiencing violence (*n* = 447) and the perceived helpfulness of supports accessed.

Of the 447 women who reported help-seeking to cope with experiences of violence, the most frequently accessed supports were health care providers (*n* = 313, 70%), family/friends (*n* = 244, 55%), and other non-HIV community organizations (*n* = 235, 53%; see Figure 1). The majority of women reported high levels of perceived helpfulness across supports, with traditional justice having the lowest perceived helpfulness (82% of women who accessed traditional justice found it helpful), and peer support having the highest perceived helpfulness among those who accessed it (96%).

**Figure 2.**
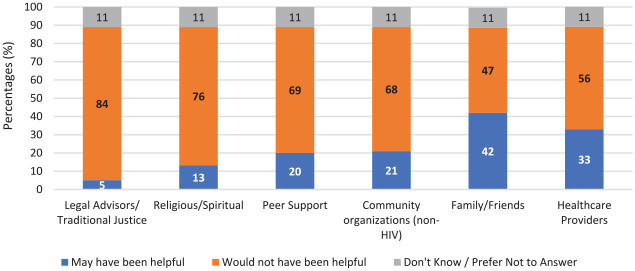
Perception of supports that might have been helpful in coping with experiences of violence, among women who did not seek help (*n* = 610).

Of the 610 women who did not report seeking help, 42% (*n* = 259) identified that family/friends may have been helpful in coping with their experiences of violence, followed by health care providers (*n* = 201, 33%), and other non-HIV community organizations (*n* = 128, 21%; see Figure 2). However, there was not one type of support identified that would have been particularly helpful in coping with their experiences. Overall, approximately one quarter (*n* = 163) reported that no supports would have been helpful in coping with their experiences of violence.

## Discussion

Despite an alarmingly high proportion of women living with HIV enrolled in CHIWOS reporting experiencing violence as an adult (80%), less than half sought help to cope with their experience. In adjusted analyses, we found that women in Ontario were less likely to seek help compared to women in British Columbia, with no other significant provincial differences. Women who were ever diagnosed with a mental health condition, reported a history of injection drug use, experienced violence as a child, and who reported experiencing sexism in their daily lives were *more likely* to seek help. Resilience was not a significant predictor of help-seeking after violence, which suggests the importance of external factors in shaping help-seeking patterns rather than an intrinsic personal motivation or ability. While our findings contrast with previous research suggesting that women who experience multiple forms of oppression and discrimination in their daily lives are less likely to seek help (Einboden & Varcoe, 2019), they resonate with a hypothesis that women who are already connected into care through their health care providers, informal networks, and other community organizations are more likely to seek help as they have trusted informal and/or formal relationships to rely on (Barrett & St Pierre, 2011). For example, women who have a history of injection drug use may be connected with other services, including harm reduction or mental health, which may open opportunities to receive help for violence.

Previous research in a Swedish cohort similarly found that women who reported experiencing childhood violence were more likely to seek help after experiencing intimate partner violence (Popescu et al., 2010), although this connection was theorized to represent an internal resilience, which was not significant in our analysis. Furthermore, our finding that experiencing sexism predicted help-seeking may be explained by a linkage between experiencing sexism and poor mental health, prompting a previous connection to mental health care (Moradi & Funderburk, 2006); or, it may signal an understanding of violent acts in their lives and that they have less acceptance of violent and/or sexist behaviors (Rollero et al., 2019). These pathways that facilitate help-seeking need to be explored and clarified further.

Among women who did seek help, family and friends, health care providers, and non-HIV organizations were the most frequently accessed supports. However, regardless of the support that was accessed, women reported high levels of perceived helpfulness. Women who did not seek help largely did not identify that any particular support would have been helpful, although almost half (42%) thought family/friends might have been helpful in coping with their experiences. Approximately one quarter reported that they did not think any supports would have been helpful in coping with their experiences of violence.

The Canadian Violence Against Women Survey found much higher rates of help-seeking among Canadian women, with 76% seeking at least one source of support after experiencing violence (Kaukinen, 2002). However, help-seeking needs to be understood within the larger social context in which an individual is situated. The economic dependency hypothesis suggests that one barrier to help-seeking, especially in instances where the perpetrator of violence is a family member or intimate partner, relies on the socio-economic standing of the woman herself (Liang et al., 2005). Some women may be financially dependent on their partner or caregiver who is perpetrating the violence; or, due to classist biases in service provision, women who are living in poverty may not have trusting relationships with existing services (Liang et al., 2005). However, our study suggests that women living with HIV reporting a higher annual income were less likely to seek help. This may be explained by our dichotomization of annual income by $20,000 CAD, obscuring potential patterns among high-, middle-, and low-income households. Women reporting a higher income may also believe that they have the resources available to deal with the violence, rather than seeking outside help; or, they may fear consequences to their economic status by seeking help (Kaukinen et al., 2013).

Indigenous women living with HIV were more likely to seek help compared to white women in our univariable analysis. This may be due to the ongoing activism of Indigenous peoples to reclaim stolen culture, spirituality, and land (Barker, 2015), thereby creating opportunities to seek and receive help if needed (Lavallee & Poole, 2010). Women who identified as African, Caribbean, or Black were less likely to seek help than white women, which was in contrast to previous findings that racial minority status was not a determinant of help-seeking in Canada (Hyman et al., 2009). A higher proportion of women living with HIV who are refugee/other or have permanent residency did not seek help after experiencing violence compared to Canadian citizens, which remained significant in our univariable model. Therefore, a potential mediating factor could be precarious legal status in Canada, deterring women from seeking help in fear of jeopardizing their legal status. Fear of having culturally insensitive care or being stereotyped (Ghafournia & Easteal, 2019; Monterrosa, 2019) may further discourage women from speaking out (Cho et al., 2020). Ultimately, as neither variable was selected for inclusion in the multivariable model, and we may have an underrepresentation of African, Caribbean, and Black women in this analysis (see Supplementary File 1), further investigation into legal barriers that may be deterring women from accessing services is needed.

We found that a high proportion of women who are food insecure, living in poverty, and facing other sources of discrimination sought help, indicating that services provided to women experiencing violence could incorporate some cross-services such as financial assistance or access to food banks (Postmus et al., 2009). In any service provision, it will be important to be conscious of secondary victimization and institutional violence within services and institutions and the impact of institutional barriers that result in additional trauma for women experiencing violence. This will require providers to have a nuanced understanding of the way that social and structural factors, social identities, and enabling or disabling environments shape and potentially constrain the decisions that people are able to make (Einboden & Varcoe, 2019).

### Sources of Support Accessed

Similar to our findings, previous studies looking at help-seeking patterns among women living with HIV who experienced violence have found that the most common types of supports accessed were family members and their HIV or health care providers (Illangasekare et al., 2014). When engaged in HIV care, women living with HIV will consult a health care provider multiple times a year, providing a potential connection to seeking help (Illangasekare et al., 2014). For some women living with HIV, developing strong social networks was imperative in coping with their HIV diagnosis (Derlega et al., 2002; Peterson, 2010). Therefore, women who already have strong networks in place in coping with their diagnosis may have pre-existing connections and networks that they can lean on. However, the stigma of living with HIV can also limit social support (Smith et al., 2008) and act as a deterrent to help-seeking (Hudson et al., 2001), especially considering that some women experience this upon disclosure of their positive status. Fear of disclosure may impede access to peer support services designed for people living with HIV. The visibility of walking into an organization for people living with HIV, or accessing services from an HIV-specific organization or program, may cause involuntary disclosure, or fear of disclosure, of their HIV status (Greene et al., 2015). Furthermore, there are limited women-specific HIV-related organizations and/or programming provided in Canada. Some women living with HIV have reported that they are not likely to seek support from an HIV-related organization if they do not see their unique needs reflected within the offered programming (Greene et al., 2015).

Consistent with our findings, accessing legal services have been perceived as less helpful in comparison to other sources of support (eg., professional counseling or self-help groups) (Einboden & Varcoe, 2019; Kaukinen et al., 2013; Postmus et al., 2009). This is unsurprising, as there may be mistrust with the legal system, especially for women who are Indigenous, racialized, or use substances (Einboden & Varcoe, 2019). This may be a particularly salient issue for women living with HIV, where threats of HIV nondisclosure criminalization could keep women living with HIV in abusive relationships or violent situations (Greene et al., 2019), and potentially discourage them from sharing openly with their health care provider (Greene et al., 2019; Patterson et al., 2019) due to the fear of HIV nondisclosure prosecution.

### Systems Impeding the Ability to Seek Help

There are many reasons why a woman may not have accessed help. Some women may not be aware of any sources of support available to them (Hyman et al., 2009), do not feel like they want or need help, believe that the violence they experienced was not “severe” enough (Hyman et al., 2009; Illangasekare et al., 2014), or want to “take care of the problem themselves” (Postmus et al., 2009). Some women report fearing potential repercussions of seeking help, such as the involvement of child welfare services (Peterson, 2010). Especially after considering the history of mistreatment and systematic oppression of women living with HIV, Indigenous women, and racialized women throughout the Canadian health care (Mill et al., 2009; Phillips-Beck et al., 2020; Wagner et al., 2016), social services (Greene et al., 2014; Martens, 2019), and legal system (Cameron, 2009; Wortley & Owusu-Bempah, 2012), this is not a surprising deterrent to accessing care in a particularly vulnerable time.

Other barriers to accessing social support from providers and their social networks include: fear of abandonment; a lack of support services available; a mismatch in goals between the woman and the person she is seeking support form (eg., she is seeking emotional support, but the supporter provides informational support instead) (Derlega et al., 2002); fear of burdening their families and friends with disclosures of violence and HIV (Peterson, 2010); or receiving judgment (Illangasekare et al., 2014). Disclosure of HIV status was highlighted as a particularly important barrier to accessing social support and a lack of resources available specifically for women living with HIV (Peterson, 2010), especially for aging women living with HIV who are largely ignored in interventions for gender-based violence (Cooper & Crockett, 2015).

### Moving Beyond a Focus on the Individual

When addressing help-seeking for women living with HIV, the underlying values, priorities, and histories within society need to be addressed. Care models must engage with dismantling stigma, oppression, and social inequities that are influencing health outcomes. Our analysis highlighted health care providers and non-HIV specific organizations as particularly important and helpful formal supports for women living with HIV who have experienced violence. Therefore, ensuring that those who provide care or services to women—whether in a formal or informal setting—are equipped to explore issues of safety in a supportive, empowering, and nonjudgmental manner is particularly vital.

Implementing trauma-aware practices and engraining women-centered principles may create an opportunity for women to feel safe reaching out; and for women who are already connected into care for their HIV treatment, health clinics and other points of care may be the optimal starting point (Decker et al., 2016; Dhairyawan et al., 2013). Supports offered should be flexible—including options to meet with peers—as well as responsive to practical barriers, such as child care commitments and travel costs (Wielding & Scott, 2016). In building trauma-aware care models, the focus centers on creating an organizational culture that is safe for everyone, including staff, regardless of disclosure of violence. In this “universal precautions” approach, staff and providers are trained and supported to provide emotionally, physically, and culturally safe care to all people (Einboden & Varcoe, 2019).

Dismantling harmful societal views outside of the clinic or organizational setting must also be a priority, to raise awareness, dispel stigma, and correct misassumptions that exist. One such model is outlined in the gender-based violence and recovery center at Coast Provincial General Hospital in Mombasa, Kenya (Temmerman et al., 2019) and at Oak Tree Clinic at the British Columbia Women’s Hospital in Vancouver, British Columbia (Oak Tree Clinic, 2014). Both care models provide competent medical care, and seek to respond to the societal and structural basis of gender-based violence by educating health care and service providers in surrounding regions and advocating for this training to be incorporated into medical curricula (Kestler et al., 2018; Temmerman et al., 2019).

Ultimately, in all discussions of supporting women who have experienced violence, it is important to balance individual needs with larger social responses that perpetuate and sustain high patterns of violence against women living with HIV. Supporting women to cope with their experiences is an overly individualistic approach to a structural, pervasive public health and safety issue. Strategies must support women who are seeking help after experiences of violence, while minimizing risk of future harm and responding with “autonomy, confidentiality, safety, and dignity” (Einboden & Varcoe, 2019). This should include review of institutional policies, and a critique of the broader social and political climate that normalize violence against women, especially women who live within the intersections of poverty, HIV, and racialization.

### Limitations

There are important limitations of this study. With a cross-sectional analysis, we are only able to assess associations of women who report ever-experiencing violence as an adult, and we are not able to assess temporality or causality of its covariates, including assessing the time between the experience of violence and when support was accessed. Furthermore, we were not able to assess who perpetrated the violence, which previous research has found to have a significant predictor effect of help-seeking (Kaukinen, 2002), nor why a particular support was helpful or unhelpful, nor the frequency with which the source of support was accessed. Furthermore, asking about accessing sources of support in our survey has an implicit assumption that services sought are available and accessible (Postmus et al., 2009). Therefore, we are also not able to understand if women did not seek help because they did not want to, or because it was not accessible.

We were not able to tease out if a particular source of support was particularly helpful for coping with certain experiences of violence. The type and severity of intimate partner violence (non-HIV specific) experienced or the consequence of the violence (e.g., PTSD) (Cho et al., 2020) may predict which services women access.

In a supplementary analysis, we found a higher proportion of African, Caribbean, and Black women, women who reported an education lower than high school, and women with more than two dependents chose not to complete the violence section and were excluded (see Supplementary File 1). We anticipate that women chose not to complete the violence section to avoid answering emotionally activating questions about violence they experienced in the past, and thus have an underrepresentation of both groups in our prevalence estimates of how many women living with HIV experience violence in adulthood, and in our analysis. Although ethnicity was not included in our adjusted analyses, bivariate and univariate analyses show that there may be important concerns for racialized women living with HIV in accessing help, which will need to be explored further.

Finally, there are always limitations regarding overall study recruitment. CHIWOS’ peer-driven recruitment strategies, including support from community partners, were effective in recruiting women living with HIV from diverse social positions (e.g., ethnicity, sexual orientation, experiences with injection drug use) (Webster et al., 2018). However, we were limited in recruiting younger women, women with a more recent HIV diagnosis, and women who may not be publicly disclosed and/or fear disclosing their HIV status for a research project (Webster et al., 2018).

Despite these limitations, in recognizing the multitude of violence that women living with HIV report experiencing, this analysis still illustrates an important relationship between women who are seeking help and covariates that may help to inform existing services to support women who experience violence in the future.

## Conclusion

Our analysis illuminates a troubling gap whereby women living with HIV are experiencing high levels of violence, yet less than half report seeking formal or informal support, despite a high level of perceived helpfulness among women who did seek help after experiencing violence. Implementing women-centered and trauma-aware care models designed by, with, and for women living with HIV and providers who care for them in any setting where women access care could help to mediate experiences of violence and create open communication between women and their providers (Women and HIV Research Program at Women’s College Hospital, 2020a, 2020b). Further research into understanding the barriers and facilitators to accessing care are critical.

## Supplemental Material

sj-docx-1-vaw-10.1177_10778012211019047 – Supplemental material for Help-Seeking to Cope With Experiences of Violence Among Women Living With HIV in Canada Supplemental material, sj-docx-1-vaw-10.1177_10778012211019047 for Help-Seeking to Cope With Experiences of Violence Among Women Living With HIV in Canada by Rebecca Gormley, Valerie Nicholson, Rebeccah Parry, Melanie Lee, Kath Webster, Margarite Sanchez, Claudette Cardinal, Jenny Li, Lu Wang, Rosa Balleny, Alexandra de Pokomandy, Mona Loutfy and Angela Kaida in Violence Against Women
