# Supplementary material for: Help-Seeking to Cope With Experiences of Violence Among Women Living With HIV in Canada
Source: Violence Against Women. 2021 Jul 16;28(3-4):823–50. doi: 10.1177/10778012211019047 (PMC8785290; doi:10.1177/10778012211019047)
Supplement: sj-docx-1-vaw-10.1177_10778012211019047 – Supplemental material for Help-Seeking to Cope With Experiences of Violence Among Women Living With HIV in Canada [file sj-docx-1-vaw-10.1177_10778012211019047.docx]

Supplementary File 1. Sociodemographic and psychosocial characteristics of women living with HIV enrolled in CHIWOS who completed the violence section (n=1,057) and who did not complete the violence section (n=105) and were thus excluded from the analysis (row %).

| **Variable** | **Completed Violence Section** | | | |  |
| --- | --- | --- | --- | --- | --- |
|  | **Yes** | | **No** | |  |
|  | (n=1057) | | (n=105) | |  |
|  | n (row %) or median [IQR] | | n (row %) or median [IQR] | | P-value |
| **Province of interview** |  |  |  |  | 0.409 |
| British Columbia | 316 (92.1) | | 27 (7.9) | |  |
| Ontario | 472 (89.7) | | 54 (10.3) | |  |
| Quebec | 269 (91.8) | | 24 (8.2) | |  |
|  |  |  |  |  |  |
| **Age** | 43 [36-50] | | 43 [36-51] | | 0.935 |
|  |  |  |  |  |  |
| **Ethnicity** |  |  |  |  | <0.001 |
| Indigenous | 247 (89.8) | | 28 (10.2) | |  |
| African/Caribbean/Black | 273 (85.8) | | 45 (14.2) | |  |
| White | 456 (94.2) | | 28 (5.8) | |  |
| Mixed Race / Other | 81 (95.3) | | 4 (4.7) | |  |
|  |  |  |  |  |  |
| **Legal status in Canada** |  |  |  |  | 0.106 |
| Canadian citizen | 870 (91.8) | | 78 (8.2) | |  |
| Landed/permanent resident | 111 (87.4) | | 16 (12.6) | |  |
| Refugee/Other | 72 (86.7) | | 11 (13.3) | |  |
| DK/PNTA | 4 (100.0) | |  | |  |
|  |  |  |  |  |  |
| **Gender identity** |  |  |  |  | 0.809 |
| Woman | 1009 (91.0) | | 100 (9.0) | |  |
| Transwoman/Two-  Spirited/Queer/Other | 48 (90.6) | | 5 (9.4) | |  |
|  |  |  |  |  |  |
| **Sexual orientation** |  |  |  |  | 0.240 |
| Heterosexual | 909 (90.5) | | 95 (9.5) | |  |
| LGBTQ | 143 (93.5) | | 10 (6.5) | |  |
| DK/PNTA | 5 (100.0) | |  |  |  |
|  |  | |  |  |  |
| **Household yearly income** |  |  |  |  | 0.219 |
| < $20000 | 681 (90.4) | | 72 (9.6) | |  |
| ≥$20000 | 352 (92.6) | | 28 (7.4) | |  |
| DK/PNTA | 24 (82.8) | | 5 (17.2) | |  |
|  |  | |  | |  |
| **Housing stability** |  |  |  |  | 0.482 |
| Stable | 943 (90.8) | | 96 (9.2) | |  |
| Unstable | 114 (92.7) | | 9 (7.3) | |  |
|  |  |  |  |  |  |
| **Education** |  |  |  |  | 0.021 |
| Lower than high school | 178 (86.8) | | 27 (13.2) | |  |
| High school or higher | 875 (91.9) | | 77 (8.1) | |  |
| DK/PNTA | 4 (80.0) | | 1 (20.0) | |  |
|  |  |  |  |  |  |
| **Current sex work** |  |  |  |  | 0.773 |
| No | 938 (95.7) | | 42 (4.3) | |  |
| Yes | 76 (95.0) | | 4 (5.0) | |  |
| DK/PNTA | 43 (42.2) | | 59 (57.8) | |  |
|  |  | |  | |  |
| **Injection drug use (ever)** |  |  |  |  | 0.829 |
| No | 651 (91.2) | | 63 (8.8) | |  |
| Yes | 390 (91.5) | | 36 (8.5) | |  |
| DK/PNTA | 16 (72.7) | | 6 (27.3) | |  |
|  |  |  |  |  |  |
| **Incarceration** |  |  |  |  | 0.067 |
| Never | 596 (90.6) | | 62 (9.4) | |  |
| Ever (but not last year) | 384 (92.8) | | 30 (7.2) | |  |
| Recent (within the last year) | 74 (85.1) | | 13 (14.9) | |  |
|  |  |  |  |  |  |
| **Number of dependents** |  |  |  |  | 0.007 |
| 0 | 611 (93.0) | | 46 (7.0) | |  |
| 1 | 206 (91.2) | | 20 (8.8) | |  |
| ≥2 | 238 (86.5) | | 37 (13.5) | |  |
| DK/PNTA | 2 (50.0) | | 2 (50.0) | |  |
|  |  |  |  |  |  |
| **Food security** |  |  |  |  | 0.393 |
| Food secure | 368 (92.0) | | 32 (8.0) | |  |
| Food insecure | 685 (90.5) | | 72 (9.5) | |  |
| DK/PNTA | 4 (80.0) | | 1 (20.0) | |  |
|  |  |  |  |  |  |
| ***Psychosocial factors*** |  |  |  |  |  |
| **Mental health condition** |  |  |  |  | 0.128 |
| No | 554 (89.9) | | 62 (10.1) | |  |
| Yes | 493 (92.5) | | 40 (7.5) | |  |
| DK/PNTA | 10 (76.9) | | 3 (23.1) | |  |
|  |  | |  | |  |
| **Resilience** s**cale** | 64 [58-68] | | 63 [59-69] | | 0.935 |
|  |  |  |  |  |  |
| **Everyday racism scale** | 17 [8-29] | | 20 [8-32] | | 0.119 |
|  |  |  |  |  |  |
| **Everyday sexism scale** | 19 [11-28] | | 17 [8-29] | | 0.594 |
|  |  |  |  |  |  |
| **HIV stigma scale** | 58 [43-70] | | 58 [45-75] | | 0.598 |
|  |  |  |  |  |  |
| ***Medical Information*** |  |  |  |  |  |
|  |  |  |  |  |  |
| **Years living with HIV** |  |  |  |  | 0.597 |
| Less than 6 years | 242 (92.4) | | 20 (7.6) | |  |
| 6 to 14 years | 413 (90.2) | | 45 (9.8) | |  |
| More than 14 years | 369 (91.3) | | 35 (8.7) | |  |
| DK/PNTA | 33 (86.8) | | 5 (13.2) | |  |
|  |  |  |  |  |  |
| **Current ARV use** |  |  |  |  | 0.796 |
| Currently on ARVs | 905 (91.0) | | 90 (9.0) | |  |
| Not currently but previously | 53 (93.0) | | 4 (7.0) | |  |
| Never on ARVs | 97 (89.8) | | 11 (10.2) | |  |
| DK/PNTA | 2 (100.0) | |  |  |  |
